# The gut microbiome and cross-reactivity of food allergens: current understanding, insights, and future directions

**DOI:** 10.3389/falgy.2024.1503380

**Published:** 2025-01-13

**Authors:** Carolina Taico Oliva, Ibrahim Musa, Daniel Kopulos, Fariba Ardalani, Anish Maskey, Aaron Wilson, Nan Yang, Xiu-Min Li

**Affiliations:** ^1^ Department of Pathology, Microbiology & Immunology, New York Medical College, Valhalla, NY, United States; ^2^ General Nutraceutical Technology LLC, Elmsford, NY, United States; ^3^ Department of Otolaryngology, School of Medicine, New York Medical College, Valhalla, NY, United States; ^4^ Department of Dermatology, School of Medicine, New York Medical College, Valhalla, NY, United States

**Keywords:** gut microbiome, cross-reactivity, probiotics, IgA, short chain fatty acids

## Abstract

This mini-review examines the emerging role of the gut microbiome in influencing food allergen cross-reactivity. It specifically focuses on how microbial diversity, antigens, and metabolites impact IgE-mediated allergic responses. Cross-reactivity occurs when structurally similar food and microbial antigens trigger hypersensitivities, affecting millions of people worldwide. Recent research underscores the significance of microbial diversity in early life for developing immune tolerance. Beneficial strains, such as Lactobacillus and Bifidobacterium, play a crucial role in supporting the functions of T regulatory cells (Tregs) and immunoglobulin A (IgA). Additionally, we discuss microbial metabolites, particularly short-chain fatty acids (SCFAs), which enhance immune tolerance by promoting Treg differentiation and maintaining gut barrier integrity, thereby reducing allergen entry. However, it is important to note that SCFAs can provoke inflammatory responses under certain conditions, highlighting the necessity for targeted research on their dual effects. Dysbiosis-related intestinal permeability, often referred to as “leaky gut,” can further worsen cross-reactivity. Microbial antigens like lipopolysaccharides (LPS) are known to influence Th2-dominant responses.

## 1 Introduction

Food allergies affect approximately 32 million people in the United States, impacting both children and adults (1). These allergies often involve immune cross-reactivity, where the immune system mistakenly responds to allergens that are structurally similar. This occurs mainly because of shared amino acid sequences or molecular structures among different allergens, resulting in IgE-mediated responses that complicate management for those affected (2, 3). Significant progress has been made in understanding the mechanisms behind cross-reactivity, but recent research has also highlighted a growing interest in the role of gut microbiota in the development of allergies. In particular, changes in gut microbial composition may influence immune tolerance, affecting the likelihood of allergic reactions to certain foods.

The interplay between food allergen cross-reactivity and intestinal microbiota is increasingly recognized as a significant area of research. Cross-reactivity occurs when the immune system mistakenly targets multiple allergens due to structural similarities, often exacerbating allergic responses in individuals predisposed to hypersensitivity. This recognition is due to shared regions or sequences of amino acids known as Cross-reactivity in food allergens is believed to be triggered by a 70% similarity in the amino acid. However, the structure and overall physiochemical composition of allergens and epitopes are the primary factors behind cross-reactivity and allergies (4, 5). Gut microbiota, comprising diverse bacterial communities, is essential for immune regulation, particularly in developing tolerance against allergens. Reduced microbial diversity, especially early in life, has been linked to increased susceptibility to allergies. Certain microbes, such as *Lactobacillus* and *Bifidobacterium*, support immune tolerance by promoting the development of regulatory T cells (Tregs) and immunoglobulin A (IgA), which are essential for maintaining immune balance and preventing hypersensitivity (6, 7). A diverse microbiome is essential for training the immune system to differentiate between harmful and harmless antigens, which may reduce cross-reactivity.

Microbial antigens are molecules or fragments derived from microorganisms that stimulate an immune response (8). They are increasingly recognized as important components in immune reactions. Some microbial antigens can resemble dietary proteins, potentially affecting immune responses and triggering allergic reactions through cross-reactivity mechanisms. Bacterial lipopolysaccharides (LPS), which are components of the outer membrane of Gram-negative bacteria, are well-known microbial antigens that can activate immune cells and influence allergic responses (9).

Short-chain fatty acids (SCFAs), which are produced through microbial fermentation, play a significant role in influencing the differentiation of immune cells, especially regulatory T cells (Tregs). They do this by acetylating histones, which are crucial for regulating gene expression (10). SCFAs, along with other metabolites, help reduce inflammation and maintain the integrity of the intestinal barrier, potentially lowering the risk of allergen sensitization (11). This function is particularly important in the context of cross-reactivity, as stable immune modulation may help mitigate excessive IgE responses.

The integrity of the intestinal barrier is crucial for preventing the translocation of antigens and microbial components into the bloodstream. Dysbiosis, which refers to an imbalance in gut microbial composition, is linked to increased intestinal permeability, commonly referred to as “leaky gut.” This condition can facilitate the exposure of allergens to immune cells, potentially triggering cross-reactive immune responses. Probiotic interventions, specifically with strains like Lactobacillus rhamnosus, have shown promise in restoring barrier function and reducing sensitivity to allergens (9).

**Figure 1:**
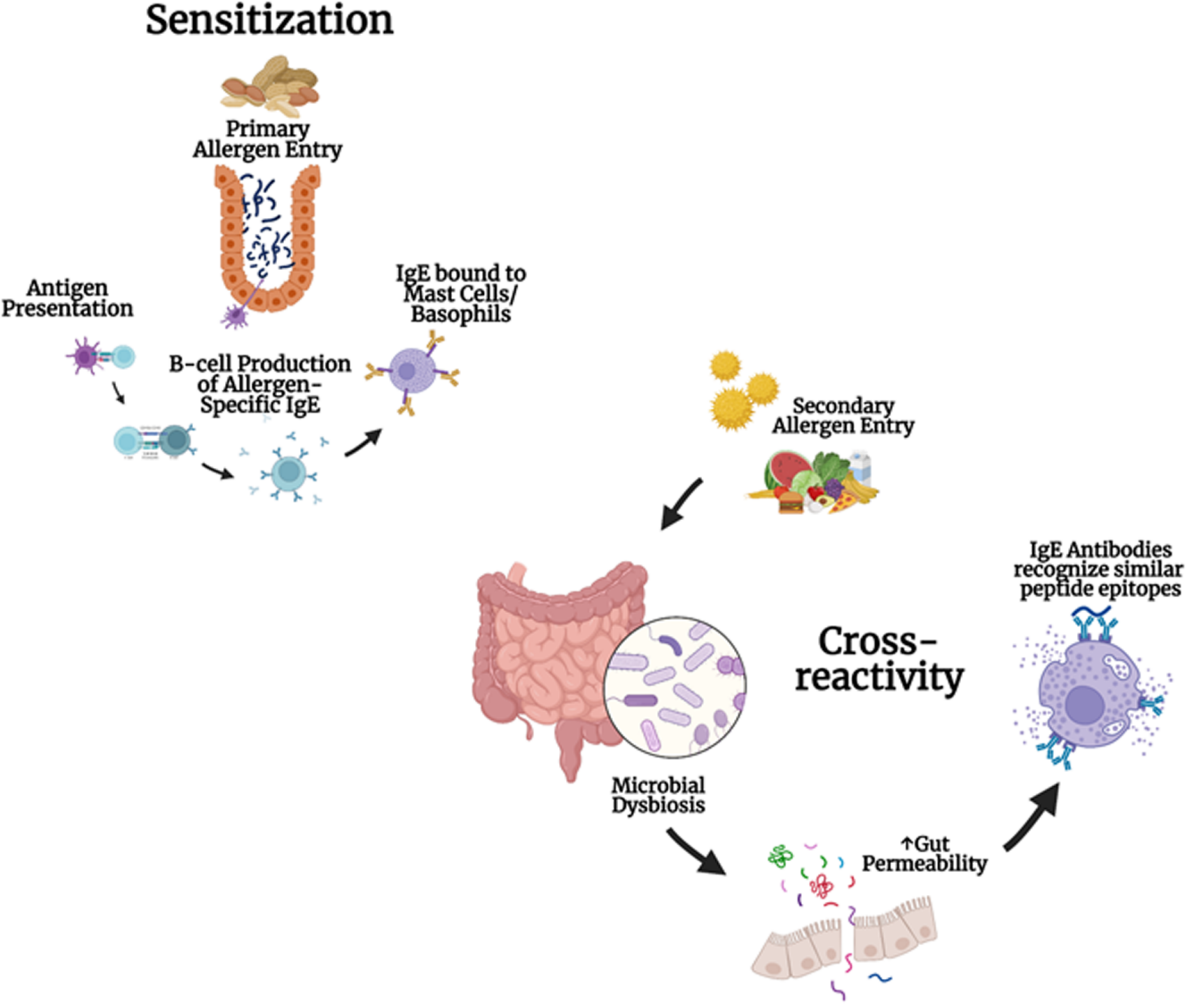
Development of cross-reactivity to multiple foods and microbial dysbiosis: cross-reactivity and allergen sensitization involve the primary entry of allergens, antigen presentation, IgE production in B cells, and the binding of IgE to mast cells and basophils. Microbial dysbiosis and gut permeability can lead to IgE antibodies recognizing similar peptide epitopes.

Understanding the complex relationship between gut microbiota and allergen cross-reactivity could lead to innovative therapeutic strategies that focus on modulating the microbiota to manage food allergies. This area is still largely unexplored, but interventions targeting the microbiome have significant potential for preventing and treating allergic reactions that result from cross-reactivity. This review evaluates the current knowledge about how gut microbiota diversity, microbial metabolites, and microbiome-targeted therapies—such as probiotics—are related to food allergen cross-reactivity. By providing a detailed analysis of microbial species, immune pathways, and possible interventions, we investigate how adjusting the gut microbiome may help decrease cross-reactivity and improve health outcomes for individuals with food allergies. Ultimately, this review aims to synthesize evidence regarding the impact of gut microbiota on food allergen cross-reactivity, offering insights into therapeutic approaches and highlighting research gaps in this emerging field (Figure 1).

## 2 Results

### 2.1 Antigen processing and molecular mimicry

The structural similarities among microbial, dietary, and environmental antigens play a significant role in cross-reactive IgE responses, often due to a phenomenon known as molecular mimicry. This occurs when the immune system detects structurally similar epitopes across different antigens. A classic example is pollen-food syndrome, where IgE antibodies produced in response to pollen proteins can also react with similar epitopes found in certain foods, resulting in allergic reactions (12, 13). Moreover, microbial peptides can imitate dietary proteins, increasing the chance of their presentation to the immune system. This can trigger allergic sensitization, especially in individuals with compromised barriers, such as atopic dermatitis. This pathway emphasizes the intricate interactions between the gut microbiome and the immune system in fostering IgE-mediated cross-reactivity. In cases of dysbiosis, excessive exposure to pathogen-associated molecular patterns (PAMPs), including lipopolysaccharides (LPS), can significantly impact immune responses. LPS are identified by pattern recognition receptors (PRRs) on immune cells, leading to a skewed Th2 response that promotes IgE production and exacerbates cross-reactivity (13, 14). These findings highlight the importance of further investigating specific microbial antigens responsible for triggering cross-reactive IgE responses and their potential role in the progression of allergic diseases. Such research could pave the way for developing microbiome-targeted therapies to address these issues (13).

### 2.2 Influence of early-life microbial exposure on cross sensitization

Early microbial colonization is crucial for immune development, particularly in establishing oral tolerance, which allows the immune system to differentiate harmful from harmless antigens. Exposure to a diverse microbiome during infancy aids in developing regulatory T cells and enhances IgA production, both essential in maintaining immune balance. Factors such as vaginal delivery and breastfeeding are associated with colonization by beneficial microbes like Bifidobacterium and Lactobacillus, which can help mitigate allergic sensitization (6).

Conversely, antibiotic exposure or C-section delivery disruptions may reduce microbial diversity, increasing the risk of immune dysregulation and hypersensitivity later in life. This is especially critical during the early developmental window, where dysbiosis can lead to a weakened gut barrier and elevated risk of allergic diseases, including food allergies, asthma, and atopic dermatitis (6, 15). Future studies should focus on identifying specific microbial species and their long-term influence on immune tolerance, which may have significant implications for allergy prevention and early-life interventions.

### 2.3 Microbial metabolites and immune regulation

Short-chain fatty acids (SCFAs), produced through microbial fermentation, are crucial in supporting immune tolerance by promoting the differentiation of regulatory T cells (Tregs) and modulating inflammation (16). SCFAs achieve this by acetylating histones on regulatory genes, including Foxp3, which is essential for Treg differentiation. This mechanism illustrates how the gut microbiome, via SCFAs, influences immune regulation and may impact cross-reactivity. However, SCFAs can also trigger inflammatory responses under certain conditions, particularly when exposed to antigens that activate Th1 and Th17 cells (17, 18). Understanding the circumstances under which SCFAs induce either pro-inflammatory or anti-inflammatory responses is essential to comprehend their role in allergen cross-reactivity. Future research should investigate the broader range of microbial metabolites that affect immune regulation and cross-reactivity. SCFAs alone do not fully capture the entire spectrum of immune effects mediated by the gut microbiome.

### 2.4 Immune system modulation by the gut microbiome

Foxp3+ T regulatory (Treg) cells maintain immune tolerance to dietary antigens and the gut microbiome. They work closely with immunoglobulin A (IgA) to prevent exaggerated immune responses. A recent study demonstrated that deleting the Foxp3 gene in mice reduced Clostridia levels, highlighting the role of Treg cells in sustaining populations of microbes that promote Treg development (19). This balance between Tregs and T helper 2 (Th2) responses—both associated with IgE-mediated allergic reactions—is influenced by microbial diversity. Elevated levels of IgE observed in gluten-free rats suggest a potential link between dysbiosis, IgE responses, and cross-reactivity (20). This connection may guide the development of future therapies to restore microbial diversity and the balance of Tregs in individuals with food allergies. Additionally, oral administration of short-chain fatty acids (SCFAs) has shown promise in reducing systemic and local allergic responses, indicating a non-Treg-mediated pathway for modulating hypersensitivity (21). Further research is needed to determine whether other microbiome-targeted interventions, such as IgA-based therapies, can help reduce allergen cross-reactivity.

### 2.5 Gut barrier integrity and cross-reactivity

Dysbiosis, an imbalance in the gut microbiome, has been linked to increased intestinal permeability, often referred to as “leaky gut” (22). This condition allows bacterial by-products and endotoxins to enter the bloodstream. When the gut barrier is compromised, it can contribute to the development of various autoimmune and allergic conditions. Additionally, long-term exposure to dietary allergens in individuals with dysbiosis may lead to a continuously weakened gut barrier, which further heightens the risk of cross-reactivity. Probiotic interventions, such as Lactobacillus rhamnosus GG (LGG), have shown promise in restoring the integrity of the intestinal barrier by enhancing the expression of ZO-1, a marker associated with tight junctions in the gut epithelium (23). Research indicates that therapies targeting microbiomes may also influence IgA levels, potentially providing extra protection against allergen cross-reactivity (24). However, further studies are needed to better understand the effectiveness of these therapies in reducing cross-reactivity and improving gut barrier function.

## 3 Discussion

Our review highlights the increasing interest in understanding how the gut microbiome influences food allergen cross-reactivity. It emphasizes the potential for novel microbiome-targeted therapies to enhance management strategies for food allergies. The introduction pointed out the limitations of current therapeutic options for addressing cross-reactivity and underscored the significant role of the gut microbiome in immune regulation. Building on these points, our discussion now integrates findings from recent studies that explore the immune and microbial mechanisms underlying cross-reactivity, paving the way for targeted therapeutic interventions.

A critical insight from this review is the complex relationship between the gut microbiome and immune tolerance, particularly in shaping IgE-mediated responses. As outlined in the introduction, certain bacteria, such as Lactobacillus and Bifidobacterium species, are linked to immune balance through mechanisms involving regulatory T cells (Tregs) and immunoglobulin A (IgA) production. Our results indicate that early exposure to these beneficial bacteria—often through natural colonization processes during infancy, such as vaginal delivery and breastfeeding—promotes immune resilience and reduces the risk of hypersensitivities. This context helps explain why microbial dysbiosis, often resulting from factors like antibiotic use or cesarean delivery, may predispose individuals to food allergies and cross-reactivity. Future studies should focus on identifying specific microbial strains and their functional roles in immune regulation, particularly within early-life microbiomes, to develop preventive measures against allergies.

Another major theme is the role of microbial metabolites, such as short-chain fatty acids (SCFAs), in modulating immune responses that could impact allergen cross-reactivity. SCFAs, including butyrate, acetate, and propionate, are produced through the microbial fermentation of dietary fibers. These metabolites promote Treg differentiation by acetylating histones in immune cells and help maintain gut barrier integrity, preventing the translocation of allergens and microbial antigens into circulation. Our results highlighted that under certain conditions, SCFAs could also activate pro-inflammatory responses, particularly through Th1 and Th17 cell pathways. This dual nature of SCFAs underscores the need for further investigation to understand under what conditions these metabolites contribute to immune tolerance vs. sensitization. Identifying optimal concentrations and microbial species that can reliably produce beneficial SCFA profiles may inform therapeutic interventions to mitigate cross-reactivity.

Additionally, the integrity of the gut barrier plays a central role in preventing allergen sensitization. As we discussed, dysbiosis can lead to increased intestinal permeability, also known as “leaky gut,” which allows antigens and bacterial by-products to breach the epithelial barrier and interact with immune cells. This translocation can trigger heightened Th2 responses and increased IgE production, exacerbating allergic conditions. Probiotic interventions, particularly those involving Lactobacillus rhamnosus GG (LGG), have shown promise in restoring gut barrier function by upregulating tight junction proteins like ZO-1, essential for maintaining barrier integrity. These findings suggest that targeted probiotic therapies may offer a practical approach to reinforce the gut barrier, reduce allergen entry, and subsequently diminish cross-reactive responses. Further research is warranted to assess the efficacy of these therapies and to explore additional probiotic strains that may provide similar or enhanced benefits.

An intriguing area that emerged from our review involves molecular mimicry, where microbial peptides structurally resemble dietary allergens, potentially leading to IgE cross-reactivity. The results section discussed examples such as pollen-food syndrome, where IgE antibodies react to similar epitopes found in pollen and certain food proteins. This mechanism highlights the need for in-depth research into microbial epitopes and their potential role in food allergen cross-reactivity. Identifying specific microbial antigens that may trigger or exacerbate IgE-mediated responses could aid in designing precision therapies that desensitize the immune system to these cross-reactive epitopes.

Another future research direction involves exploring the diverse roles of IgA in maintaining immune tolerance and its impact on microbiome diversity and function. IgA is critical for mucosal immunity and works synergistically with Tregs to prevent inappropriate immune responses to dietary antigens. Emerging studies suggest that IgA's interactions with gut bacteria may shape microbial composition and influence immune responses, particularly in individuals with food allergies. By examining IgA-modulating therapies, future research could reveal additional mechanisms through which gut immunity can be strengthened, potentially lowering the risk of cross-reactivity in allergic individuals.
